# Predictors of pacing induced left ventricular dysfunction and cardiomyopathy assessed by three-dimensional echocardiography and speckle tracking strain

**DOI:** 10.1186/s43044-021-00136-x

**Published:** 2021-01-26

**Authors:** Moustafa Dawood, Eman Elsharkawy, Mohamed Ayman Abdel-Hay, Moustafa Nawar

**Affiliations:** Cardiology and Angiology Department, Alexandria Faculty of Medicine, Alexandria, 21568 Egypt

**Keywords:** Pacing induced LV dysfunction, Pacing-induced cardiomyopathy, 3D echocardiography, Global longitudinal strain, Cardiac pacemakers, Pacing hemodynamics

## Abstract

**Background:**

Long-term RV pacing leads to ventricular dyssynchrony, in the form of LBBB-like morphology, with subsequent detrimental effects on LV structure and function. Three-dimensional echocardiography allowed early detection of volumetric changes associated with PICMP and provided more accurate assessment of mechanical dyssynchrony. Speckle tracking strain is able to identify LV dysfunction even before any reduction in LVEF. Our aim was to study pacing effects on LV function and hemodynamics using 3D echo and speckle tracking strain.

**Results:**

This was a prospective study of 175 consecutive patients without structural heart disease (LVEF > 50%) presented for permanent pacing. Full-volume 3D echocardiography done before implantation, 1 week, and 6 months together with GLS. Patients were followed for 6 months to detect incidence of PIVD (defined as reduction in LVEF > 10% but still above 50%) and PICMP (defined as decrease in LVEF by 10% from baseline in absence of other known causes of cardiomyopathy resulting in EF< 50%). PIVD and PICMP predictors and risk factors were analyzed. Only 50 patients met study criteria. Twenty-five (50%) patients developed LV systolic dysfunction; of these, 19 (38%) developed PIVD and 6 (12%) developed PICMP. Pre-implantation GLS was significantly lower in the 6 patients who subsequently developed PICMP, compared to those who developed PIVD and the preserved EF group (mean GLS − 15.50 vs. − 21.0, − 20.0 respectively; *p* = 0.005, 0.033, respectively). At 1 week, GLS was significantly lower in the 25 patients who subsequently developed PIVD, compared to those who did not (GLS − 13.0 vs. − 18.0, respectively; *p* = 0.002). A reduction of baseline GLS by 15% or more at 1 week was associated with the development of PIVD and PICMP (*p* = < 0.001). A wider native QRS complex was associated with PIVD and PICMP (*p* = 0.008, 0.018, respectively). The other predictors were found non-significant.

**Conclusion:**

PICMP may be more common than previously reported and it may occur shortly after implantation. Pre-implantation GLS is a sensitive parameter for PICMP. One-week GLS, pre-implantation QRS complex width are early predictors for PICMP and PIVD before any reduction in EF.

**Supplementary Information:**

The online version contains supplementary material available at 10.1186/s43044-021-00136-x.

## Background

During recent years, there was a focus on the negative effects associated with long-term RV pacing. The DAVID Trial [1] and a sub-analysis of the MADIT II [2] were one of the first trials to report these changes in HF patients. It was shown in both experimental and clinical studies that RV pacing may lead to ventricular dyssynchrony, similar to that of LBBB with subsequent abnormal electrical and mechanical activation of the ventricles [3]. This results in changes in cardiac metabolism, perfusion, hemodynamics, and mechanical function [4, 5]. Long-term RV pacing may also result in structural changes and LV remodeling [6]. In addition, it has been suggested that the presence of mechanical dyssynchrony after long-term RV apical pacing is associated with reduced LV systolic function and deterioration in functional capacity [7] and in some cases adverse clinical outcomes such as atrial fibrillation, heart failure, and death [8–10]. However, in daily clinical practice, not all patients who receive RV apical pacing will experience these adverse events [11]. Initial bi-ventricular pacing prevents these complications but it cannot be applied in all cases due to its high cost and related complications.

## Aim of the work

To detect the incidence of PIVD and PICMP over 6 months post-pacing and to study the predictors and risk factors for their development.

## Methods

The study was approved by the ethics committee at our faculty of medicine. All patients provided written informed consent. Patients’ recruitment started from October 2017 to August 2018. During this period, 175 consecutive patients presented to our university hospitals for device implantation. Only 50 patients met the inclusion criteria and were included.

The exclusion criteria included the presence of more than mild valvular heart disease, left ventricular ejection fraction less than 50%, presence of ischemic heart disease, recent cardiac surgery during the last 3 months before enrollment. Patients with poor echo windows, patients with slow atrial fibrillation, or other types of arrhythmia that can affect LV function, debilitated or cancer patients with expected survival less than 1 year and patients with previously implanted devices were excluded. Of 175 patients, 125 patients were excluded. Sixty-five patients were presented with reduced LV systolic function for cardiac resynchronization therapy. Sixteen patients were assigned for ICD devices as primary or secondary prevention with no indication for permanent pacing. Twenty-two patients had previously implanted devices at the elective replacement period and were scheduled for battery replacement. Seven patients were subjected to reoperation due to device-related complications (4 with twiddler syndrome and 3 for device extraction due to bloodstream or device-related infection). Eight patients presented with slow atrial fibrillation or SSS. Two patients had poor echo views and four patients had significant valvular heart disease. One patient died before the second follow-up. The remaining 50 patients were assigned to have single-chamber (27 patients) or dual-chamber pacemaker (23 patients).

### Patient characteristics

Patient’s demographic data, medical comorbidities and indications for pacing were collected and revised 24 h before pacing. Patients’ age ranged from 12 to 97 years with mean age of 63.12 ± 16.85 years. According to gender, 27 patients were males (54%). In addition, 13 patients (26.0%) were diabetics, and 21 patients (42.0%) were hypertensive. All patients had structurally normal heart confirmed by echocardiography.

### Data collection: 2D, 3D echo full volume acquisition, and GLS analysis

A standard 12 lead ECG was done before pacing. ECG data were collected and recorded including native QRS width, axis, heart rate, degree of heart block, and presence of LBBB or RBBB-like morphology. BBB was defined according to the standard criteria. All ECG parameters were rechecked and recorded immediately after pacing and at 6 months.

A full 2D echocardiographic study was done to exclude patients with significant valvular heart disease, IHD or reduced LVEF. A 3D full volume acquisition of the left ventricle was done using the Philips Medical iE33 echocardiography system with X5-1 transthoracic probe. For 3D full volume acquisitions, ensuring adequate frame rate, packet size and capture of the full cardiac cycle, the system obtained a 30° _ 30° pyramid over 4 to 6 alternate gated cardiac cycles. The resulting dynamic 3D full volume sector was reviewed and navigated through immediately to ensure all areas of interest have been captured. All the acquisitions were ECG gated and patients were told to hold their breath during acquisition to avoid stitch artifacts. After completion of the study, 3D acquired data were transferred to Q-lab 10 for off-line analysis. In order to calculate LV volumes and EF, both the long and short LV axis were adjusted to get maximum LV dimensions and to avoid foreshortening then five landmarks were chosen to initiate edge detection by semi-automated quantification software. Four landmarks were placed at the mitral annulus and the fifth was placed at the apex in apical four or apical two views. The software delineated LV boundaries automatically, but it allowed manual modifications to include or exclude any part for more accurate adjustment of LV borders. After borders’ delineation, the software automatically calculated EDV, ESV, SV, COP, and EF.

For GLS calculation, 2D gated acquisition of the apical views (apical four, two, and three) were done according to the standard techniques. Patients were told to hold their breath during acquisition and foreshortening was avoided. GLS was calculated for each of the three apical views then mean GLS was calculated automatically. Three-dimensional echo and GLS acquisition and analysis were repeated at 1 week and 6 months after implantation to detect the incidence of PIVD (defined as a reduction in LVEF > 10 percentage points but still above 50%) and PICMP (defined as a decrease in LVEF by 10 percentage points from baseline resulting in EF < 50% (in the absence of other known causes of cardiomyopathy.

### Pacemaker implantation and programming

Implantation was performed according to the operator’s usual practice. The study population was assigned to receive a single-chamber ventricular pacemaker (23 patients) or a dual-chamber pacemaker (27 patients). Since all the previously mentioned studies showed no difference in RV lead position [12, 13], the decision was left to the operator to position the lead according to his preference. Most of the cases had RV lead in an apical position because it was easier and more readily accessible by our operators. RV apex was selected as the site of RV lead implantation in 44 candidates. The remaining six patients had septal pacing, confirmed by the post-pacing ECG axis. Patients with single chamber pacemakers were programmed to VVIR mode while those with dual-chamber pacing were programmed to DDDR mode. Rate responsive mode was selected as it resulted in better outcomes regarding patient quality of life and exercise tolerance [14]. Suggested settings for single and dual-chamber pacemakers were lower and upper rate limits of 60 beats per minute and 130 beats per minute, respectively. In order to maximize the contribution of the atrial kick to SV in the DDDR group, dynamic AV time delay [15] was selected with resting paced/sensed AV time delay adjusted to 200/150 ms [15, 16]. Pacing lowering strategies were applied to reduce ventricular pacing percentage (VP %). Patients were recruited 7–10 days post-pacing and after 6 months. Device interrogation was done to check the adjusted pacing parameters and acquire ventricular pacing percentage. Also, 3D echo and GLS were calculated at follow up visits as described before.

### Statistical analysis

The database was maintained and analyzed by an independent data-management group. To assess the distribution of the data derived from this study, we calculated the standardized skewness and kurtosis of each of the variables. Normally distributed values were expressed as mean and skewed values as the median (interquartile range). Paired two-tailed group comparisons were made with Student’s *t* (parametric) or Wilcoxon signed-rank (non-parametric) tests as appropriate. *p* values of less than 0.05 were regarded as significant.

## Results

Device interrogation showed high ventricular pacing percentage at 6 months follow-up. All the patients were pacemaker dependent with mean VP% of 92 ± 3%. There were no significant differences between both groups before implantation regarding the following parameters (ESV, EDV, SV, COP, EF, and GLS) (Table 1).

**Table 1:** Comparison between the three studied groups according to echo parameters and GLS

Predisposing factors	No decline in LVEF (***n*** = 25)		Decline in LVEF				***χ***^**2**^	^**MC**^p
			PIVD (***n*** = 19)		PICMP(***n*** = 6)			
	No.	%	No.	%	No.	%		
**EF%**								
Pre-pacing	68.0 ± 6.84		75.79 ± 5.27		68.33 ± 9.83		7.856^*^	0.001^*^
Post-pacing (at 7 days)	64.16 ± 7.64		67.74 ± 8.19		47.83 ± 6.79		15.105^*^	< 0.001^*^
Post-pacing (at 6 months)	61.76 ± 6.22		57.79 ± 4.38		36.0 ± 10.79		40.815^*^	< 0.001^*^
E**SV (ml)**								
Pre-pacing	34.12 ± 11.94		26.53 ± 8.52		34.0 ± 10.08		3.031	0.058
Post-pacing (at 7 days)	30.84 ± 10.59		28.21 ± 12.09		46.33 ± 5.82		6.562	0.003^*^
Post-pacing (at 6 months)	34.36 ± 9.81		37.53 ± 11.55		73.50 ± 35.49		16.313^*^	< 0.001^*^
**EDV (ml)**								
Pre-pacing	104.8 ± 25.81		104.7 ± 23.58		111.3 ± 25.87		0.342	0.843
Post-pacing (at 7 days)	89.40 ± 22.49		89.26 ± 22.92		93.67 ± 24.48		0.081	0.960
Post-pacing (at 6 months)	90.84 ± 22.22		89.0 ± 24.47		111.8 ± 40.35		1.222	0.543
**SV (ml)**								
Pre-pacing	70.64 ± 17.70		77.11 ± 17.32		77.33 ± 27.52		0.748	0.479
Post-pacing (at 7 days)	58.48 ± 16.12		61.05 ± 17.22		47.33 ± 19.36		1.512	0.231
Post-pacing (at 6 months)	56.44 ± 15.36		51.68 ± 14.29		38.33 ± 13.09		3.700	0.032
**COP (L/min)**								
Pre-pacing	2.96 ± 0.87		3.32 ± 1.05		2.63 ± 0.74		3.722	0.156
Post-pacing (at 7 days)	3.90 ± 0.95		4.39 ± 1.43		3.60 ± 1.21		1.715	0.424
Post-pacing (at 6 months)	3.70 ± 0.92		3.72 ± 1.38		2.72 ± 0.69		4.754	0.093
**GLS%**								
Pre-pacing	− 19.52 ± 3.62		− 20.79 ± 3.43		− 15.50 ± 2.07		5.482^*^	0.007^*^
Post-pacing (at 7 days)	− 16.80 ± 3.59		− 14.21 ± 4.21		− 10.17 ± 1.17		8.609	0.001^*^
Post-pacing (at 6 months)	− 15.40 ± 3.46		− 12.32 ± 4.60		− 8.0 ± 2.76		9.888	< 0.001^*^

*Statistically significant at *p* ≤ 0.05

**Fig. 1:**
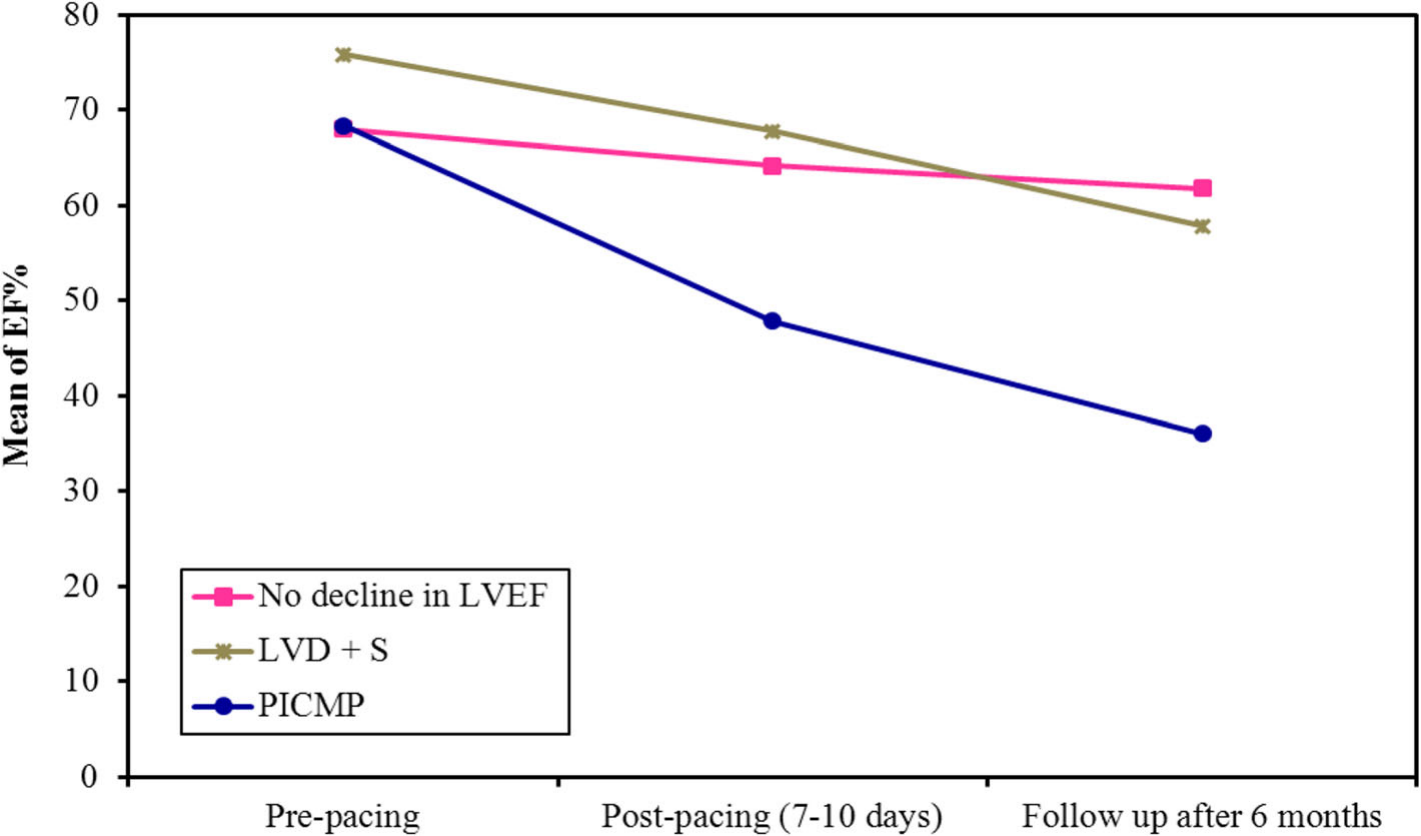
Comparison between the different periods in each of the three studied groups (no decline in LVEF, PIVD, PICMP)

Only 50 patients met the study criteria; of them, 25 patients (50%) showed a decline in post-pacing EF by 10% or more compared to baseline by the end of the study. Sub-group analysis divided them into 19 patients (38%) with PIVD and 6 patients (12%) developed PICMP (Fig. 1). In the PIVD group, mean EF% dropped from 75.8 to 57.8% at 6 months. In the PICMP group, mean EF dropped from 68.3% to 36%. Also, PICMP group showed marked increase in ESV immediately post-pacing and at 6 months (*p* = 0.003, < 0.001, respectively) with subsequent reduction in SV at 6 months (*p* = 0.032) (Table 1, Fig. 1).

**Fig. 2:**
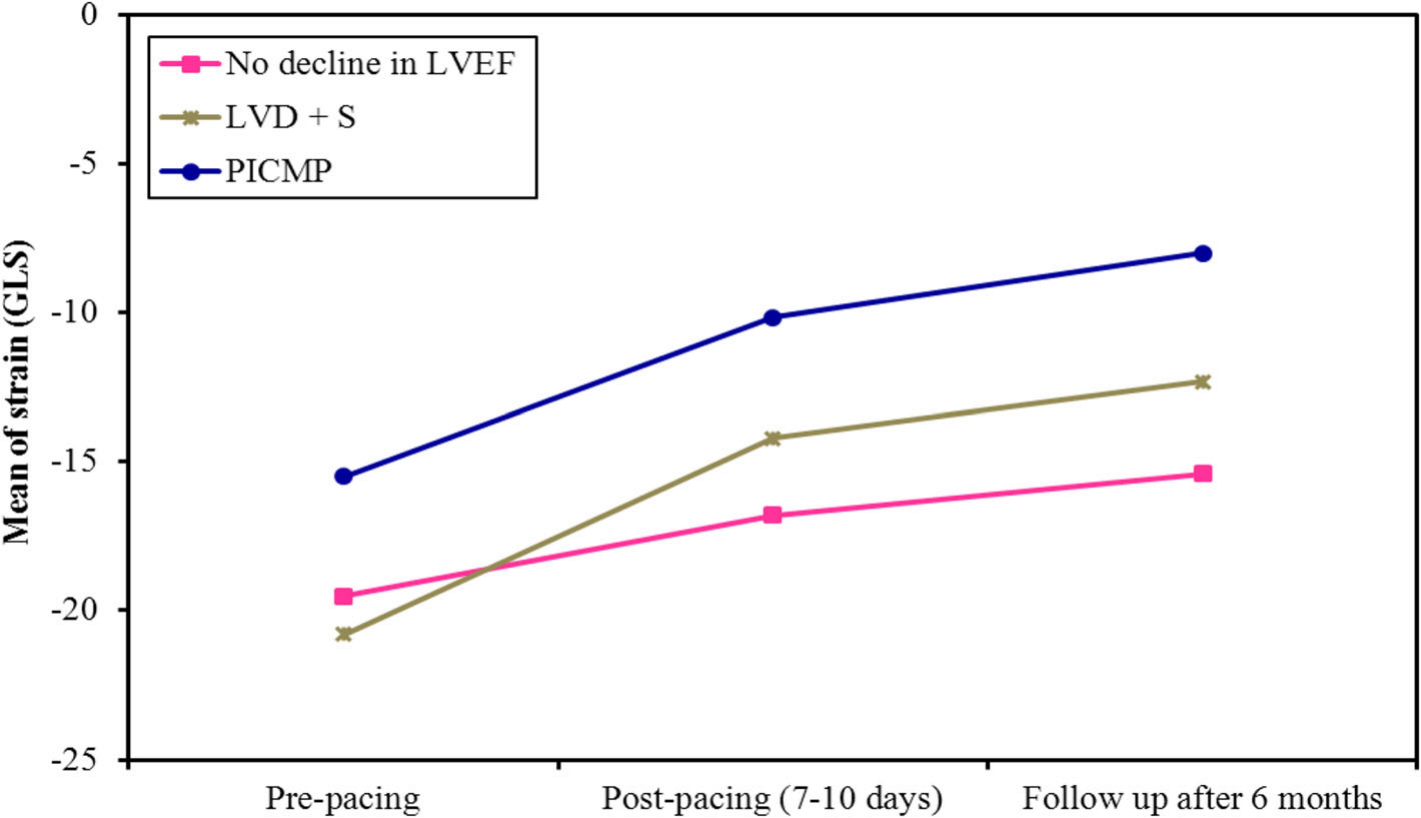
Comparison between the three studied groups according to changes in Global Longitudinal Strain (GLS) in each group

All the studied parameters were analyzed at a level of two main groups (preserved EF group and group with LV dysfunction) then repeated in a sub-group analysis between PIVD and PICMP groups. Low GLS was associated with further deterioration in LV systolic function. Pre-implantation, GLS was significantly lower in the six patients who subsequently developed PICMP, compared to those who developed PIVD and the preserved EF group (mean GLS − 15.50 vs. − 21.0, − 20.0, respectively; *p* = 0.005, 0.033, respectively), (supplementary PICMP cases 1 and 2). At 1 week, GLS was significantly lower in the 25 patients who subsequently developed LV systolic dysfunction, compared to those who did not (GLS − 13.0 vs. − 18.0 respectively; *p* = 0.002) (Table 1, Fig. 2, supplements PIVD case 1). A reduction of baseline GLS by 15% or more at 1 week was associated with the development of PIVD and PICMP (*p* = < 0.001) (Table 2). A wider native QRS complex was associated with the development of PIVD and PICMP (*p* = 0.008, 0.018, respectively). The rest of the studied parameters were found not significant (Tables 3 and 4).

**Table 2:** Comparison between the two studied groups according STS reduction percentage at 1-week follow-up visit

	Total (***n*** = 50)		No decline in LVEF(***n*** = 25)		Decline in LVEF(***n*** = 25)		Test of sig.	***p***
	No.	%	No.	%	No.	%		
**% (7 days)**								
≤ 15	23	46.0	22	88.0	1	4.0	*χ*^2^ = 29.639^*^	< 0.001^*^
> 15	27	54.0	3	12.0	24	96.0		

*χ*^2^ Chi-square test

*Statistically significant at *p* ≤ 0.05

**Table 3:** Comparison between the two studied groups according to risk factors

Predisposing factors	Total (***n*** = 50)		No decline in LVEF (***n*** = 25)		Decline in LVEF (***n*** = 25)		***χ***^**2**^	^**MC**^p
	No.	%	No.	%	No.	%		
**Age** (years)	63.12 ± 16.85		67.20 ± 18.54		59.04 ± 14.18			
Mean ± SD							202.00	0.032^*^
**Sex**								
Male	27	54.0	14	56.0	13	52.0	0.081	0.777
Female	23	46.0	11	44.0	12	48.0		
**DM**	13	26.0	7	28.0	6	24.0	0.104	0.747
**HTN**	21	42.0	14	56.0	7	28.0	4.023^*^	0.045^*^
**Device type**								
VVI	27	54.0	15	60.0	12	48.0	0.725	0.395
DDD	23	46.0	10	40.0	13	52.0		
**Lead site**								
RVA	44	88.0	22	88.0	22	83.0	0.00	1.000
RVS	6	12.0	3	12.0	3	12.0		
**Pre-existing BBB**								
No BBB	22	44.0	15	60.0	7	28.0		
RBBB	19	38.0	7	28.0	12	48.0	5.107	0.083
LBBB	9	18.0	3	12.0	6	24.0		
**QRS axis**								
N	8	16.0	4	16.0	4	16.0		
LF	38	76.0	20	80.0	18	72.0	1.514	0.883
RT	4	8.0	1	4.0	3	12.0		
**QRS width**								
**Pre-pacing**								
Mean ± SD	116.8 ± 32.27		104.6 ± 27.99		129.0 ± 32.15		178.00^*^	0.008^*^
**Post-pacing**								
Mean ± SD	151.7 ± 21.91		148.6 ± 22.98		154.8 ± 20.79		273.00	0.436
**VP%**								
**7 days**								
Mean ± SD	84.34 ± 24.83		83.40 ± 24.47		85.27 ± 25.65		285.50	0.588
**6 months**								
Mean ± SD	89.26 ± 20.62		86.80 ± 23.09		91.72 ± 17.95		289.00	0.601
**Pacing threshold**								
**7 days**								
Mean ± SD	0.57 ± 0.29		0.57 ± 0.20		0.57 ± 0.36		267.50	0.328
**6 months**								
Mean ± SD	0.88 ± 0.52		0.85 ± 0.49		0.90 ± 0.55		306.50	0.902

**Table 4:** Comparison between the three studied groups according to risk factors

Predisposing factors	No decline in LVEF (***n*** = 25)		Decline in LVEF				***χ***^**2**^	^**MC**^p
			PIVD (***n*** = 19)		PICMP(***n*** = 6)			
	No.	%	No.	%	No.	%		
**Age** (years)	67.20 ± 18.54		57.11 ± 14.77		65.17 ± 10.94			
Mean ± SD							5.565	0.062
**Sex**								
Male	14	56.0	9	47.4	4	66.7	0.790	0.786
Female	11	44.0	10	52.6	2	33.3		
**DM**	7	28.0	3	15.8	3	50.0	2.858	0.231
**HTN**	14	56.0	4	21.1	3	50.0	5.650	0.060
**Device type**								
VVI	15	60.0	10	52.6	2	33.3	1.410	0.517
DDD	10	40.0	9	47.4	4	66.7		
**Lead site**								
RVA	22	88.0	17	89.5	5	83.3	0.592	1.000
RVS	3	12.0	2	10.5	1	16.7		
**Pre-existing BBB**								
No BBB	15	60.0	7	36.8	0	0.0		
RBBB	7	28.0	8	42.1	4	66.7	8.244	0.063
LBBB	3	12.0	4	21.1	2	33.3		
**QRS Axis**								
N	4	16.0	3	15.8	1	16.7		
LF	20	80.0	13	68.4	5	83.3	3.566	0.881
RT	1	4.0	3	12.0	0	0.0		
**QRS width**								
**Pre-pacing**								
Mean ± SD	104.6 ± 27.99		125.0 ± 32.53		141.7 ± 29.94		7.848^*^	0.020^*^
**Post-pacing**								
Mean ± SD	148.6 ± 22.98		154.2 ± 19.46		156.7 ± 26.58		0.660	0.719
**VP%**								
**7 days**								
Mean ± SD	83.40 ± 24.47		81.57 ± 28.32		97.0 ± 7.35		4.136	0.126
**6 months**								
Mean ± SD	86.80 ± 23.09		89.11 ± 19.99		100.0 ± 0.0		3.848	0.146
**Pacing threshold**								
**7 days**								
Mean ± SD	0.57 ± 0.20		0.61 ± 0.41		0.46 ± 0.10		1.627	0.443
**6 months**								
Mean ± SD	0.85 ± 0.49		0.92 ± 0.62		0.83 ± 0.26		0.184	0.912

## Discussion

Our study detected a surprisingly high incidence of pacing-induced LV systolic dysfunction. In addition, it showed that it occurred shortly after implantation. Pre-implantation GLS is a sensitive parameter for PICMP. One-week GLS, pre-implantation QRS complex width are early predictors for PICMP and PIVD before any reduction in EF. Pre-implantation QRS complex width may predict the development of PICMP and PIVD.

PICMP is an important clinical problem in day-to-day practice. It was reported to develop from the first month and up to 15 years after pacemaker implantation [17]. This topic has been extensively studied previously. However, the data on the prevalence of PICMP after long-term RV pacing varies significantly. In part, this can be explained by the heterogeneity in determining the exact prevalence of PICMP:

- First, the lack of globally unified criteria of PICMP. Some studies depended on imaging modalities only for diagnosis while others added clinical endpoints. Khurshid et al. [18] defined PICMP as a decrease of 50% or more in LVEF resulting in value less than 50%. PICMP incidence as defined by an EF less than 45% was reported to be 9% after the first year [19]. Zhang et al. [20] reported new-onset heart failure in 26% prevalence of PICMP after 10 years. Kiehl et al. [21] reported a 12.3% incidence over 4.3 years. In other studies, lesser degrees of pacing-induced LV dysfunction (PIVD) have also been observed in up to two-thirds of patients with normal baseline LV function. They defined pacing-induced LV dysfunction (PIVD) as a reduction in LV ejection fraction (LVEF) by 5 percentage points or more at 12 months) [22, 23].
- Second, PM patients often suffer from co-morbidities that also cause adverse LV remodeling (e.g., IHD). A prospective study with a median follow-up of 7.8 years on 79 patients reported that (26.0%) developed new-onset HF after RV apical pacing. It has been shown that the presence of IHD was an independent risk factor for developing PICMP. A supply demand mismatch was the suggested explanation. Pacing at higher rates than the patient pre-implantation rate increases myocardial work, oxygen demand and creates relative ischemia in patients with diseased coronaries [20].
- The third reason is that not all PM patients require continuous RV pacing.

Our study tried to overcome the drawbacks of the previously reported studies and to start from they ended. Using a clinical endpoint or reporting the reduction in LVEF is often a late phenomenon. Once LVEF is decreased, despite intervention there is a failure of recovery of systolic function in up to 58% of patients [24]. This created the need for an imaging modality that is relatively inexpensive and that can pick up early PICMP.

The study was designed to implement more sensitive tools such as 3D echo and strain analysis for earlier and more accurate detection of pacing-induced negative effects on LV structure and function. We excluded patients with underlying arrhythmia, ischemic, valvular heart disease, or other potential confounding factors. Only pacing dependent patients due to advanced heart block were included.

After 6 months, pacing resulted in a significant drop in EF. This was due to pacing-induced remodeling of LV structure resulting in the expansion of LV volumes, mainly the ESV. A reduction in GLS was a predictor for further deterioration in LV systolic function. Pre-implantation, GLS was significantly lower in the six patients who subsequently developed PICMP, compared to those who developed PIVD and the preserved EF group (mean GLS − 15.50 vs. − 21.0, − 20.0, respectively; *p* = 0.005, 0.033, respectively). At 1 week, GLS was significantly lower in the 25 patients who subsequently developed LV systolic dysfunction, compared to those who did not (GLS − 13.0 vs. − 18.0, respectively; *p* = 0.002).

There is a growing number of literatures highlighting the detrimental effects of pacing on speckle tracking strain. Victoria Delgado et al. [25] studied the acute effects of RV apical pacing on LV Synchrony and Mechanics in 25 patients with structurally normal. During RV apical pacing, a more dyssynchronous LV contraction was observed together with an impairment in LV longitudinal shortening and in LV twist. In a prospective study of 36 patients with a baseline EF of more than 45% who received a permanent pacemaker followed for 6 months. A significant decrease in LV global longitudinal strain was noted in 23 (63.9%) patients by 6 months. In seven of these patients, there was a significant decrease in global longitudinal strain 24 h after implantation [24]. In a recent study conducted in 2017, 93 patients were followed for 60 ± 47 months. They found that lower LV peak GLS was the only independent predictor for LV dyssynchrony and patients with lower GLS value could be at risk of PICMP [26].

The previously published studies did not link DM or HTN to the development of PICMP. Kim et al. [27] reported abnormal post-pacing QRS axis to be associated with PICMP. Another study showed that male gender, wide native QRS complex and lower baseline LVEF to be predictors of PICMP over a period of 3.3 years [28] UK-PACE Trial [29] showed no difference between single- and dual-chamber pacemakers regarding the rates of atrial fibrillation, heart failure, or a composite of stroke, transient ischemic attack, or other thromboembolism. We studied possible risk factors for the development of PICMP. Only the width of the baseline QRS complex was found significant. This was probably due to the small sample size and short duration of follow-up. We did not include a clinical point to the trial. We divided the affected patients into two groups, PIVD and PICMP, for earlier detection. This also allowed better studying of risk factors for developing cardiomyopathy over a short- and long-term basis. Signifying the importance of baseline and post-pacing GLS as predictors for PICMP and PIVD was the main outcome of our study. Further trials with larger sample sizes and longer follow-up periods are required to validate our results and to introduce GLS in the routine follow-up of pacemaker patients.

### Study limitations

Our study had a number of limitations; the most important was the small sample size. The study duration was relatively short. We believe that higher incidence of pacing-induced LV dysfunction could be expected with longer follow up. In addition, a large number of patients received single-chamber pacing despite being in sinus rhythm. In our center, pacemakers are provided through different sources. Health assurances provide dual-chamber pacemakers for its patients if indicated. Uncovered patients are provided single-chamber devices through donations or by emergency decisions on the expense of the state as a lifesaving act. Dual-chamber pacemakers are not available in the donation sector due to financial aspects. This allows us to save a greater number of patients.

## Conclusion

Pacing-induced negative effects may be more common than previously reported and may occur shortly after implantation. This rise in its incidence is due to the application of more sensitive tools like 3D echocardiography and speckle tracking strain. Pre-implantation GLS is a sensitive parameter for the development of PICMP. One-week GLS is an early predictor for the development of PICMP and PIVD before any reduction in EF develops. Pre-implantation QRS complex width may predict the development of PICMP and PIVD.

## Supplementary Information

**Additional file 1.** PICMP case 1

**Additional file 2.** PICMP case 2

**Additional file 3.** PIVD case 1

## Data Availability

The datasets used and/or analyzed during the current study are available from the corresponding author on reasonable request.

## Abbreviations

COP: Cardiac output
EDV: End-diastolic volume
ESV: End-systolic volume
SV: Strove volume
GLS: Global longitudinal strain
LBBB: Left bundle branch block
RBBB: Right bundle branch block
LV: Left ventricle/ventricular
LVEF: Left ventricular ejection fraction
RV: Right ventricle/ventricular
PICMP: Pacing-induced cardiomyopathy
PIVD: Pacing-induced ventricular dysfunction
3D: Three-dimensional
2D: Two-dimensional
SSS: Sick sinus syndrome
ICD: Implantable cardioverter defibrillator
ECG: Electrocardiogram
IHD: Ischemic heart disease
