# Supplementary material for: Predictors of pacing induced left ventricular dysfunction and cardiomyopathy assessed by three-dimensional echocardiography and speckle tracking strain
Source: Egypt Heart J. 2021 Jan 26;73:10. doi: 10.1186/s43044-021-00136-x (PMC7838225; doi:10.1186/s43044-021-00136-x)
Supplement: Supplementary file 1 — Additional file 1. PICMP case 1 [file 43044_2021_136_MOESM1_ESM.pptx]

## Slide 1
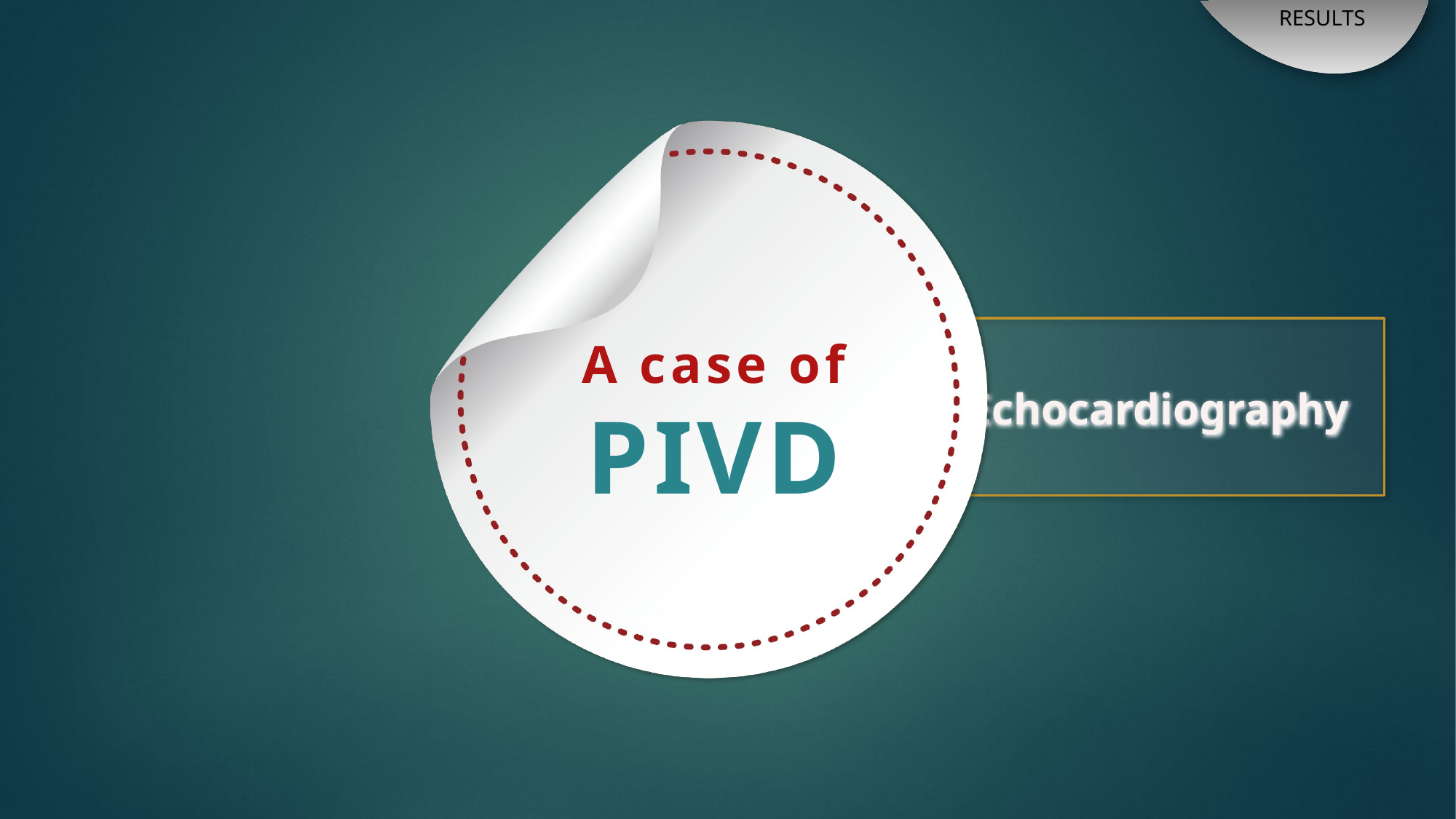

Results
A case of
PIVD
Full Volume 3D Echocardiography

## Slide 2
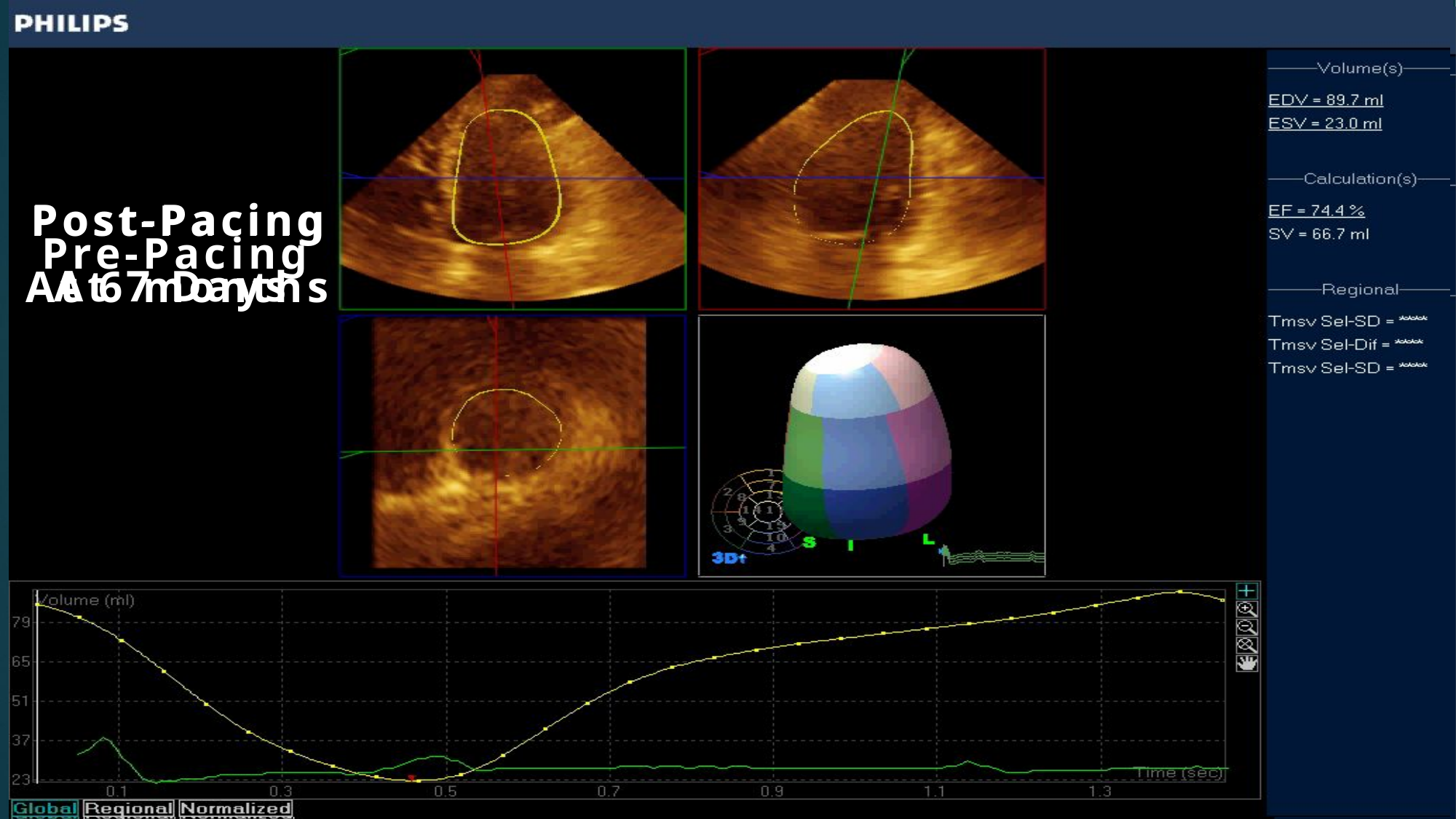

Post-Pacing
At 7 Days
Post-Pacing
At 6 months
Pre-Pacing

## Slide 3
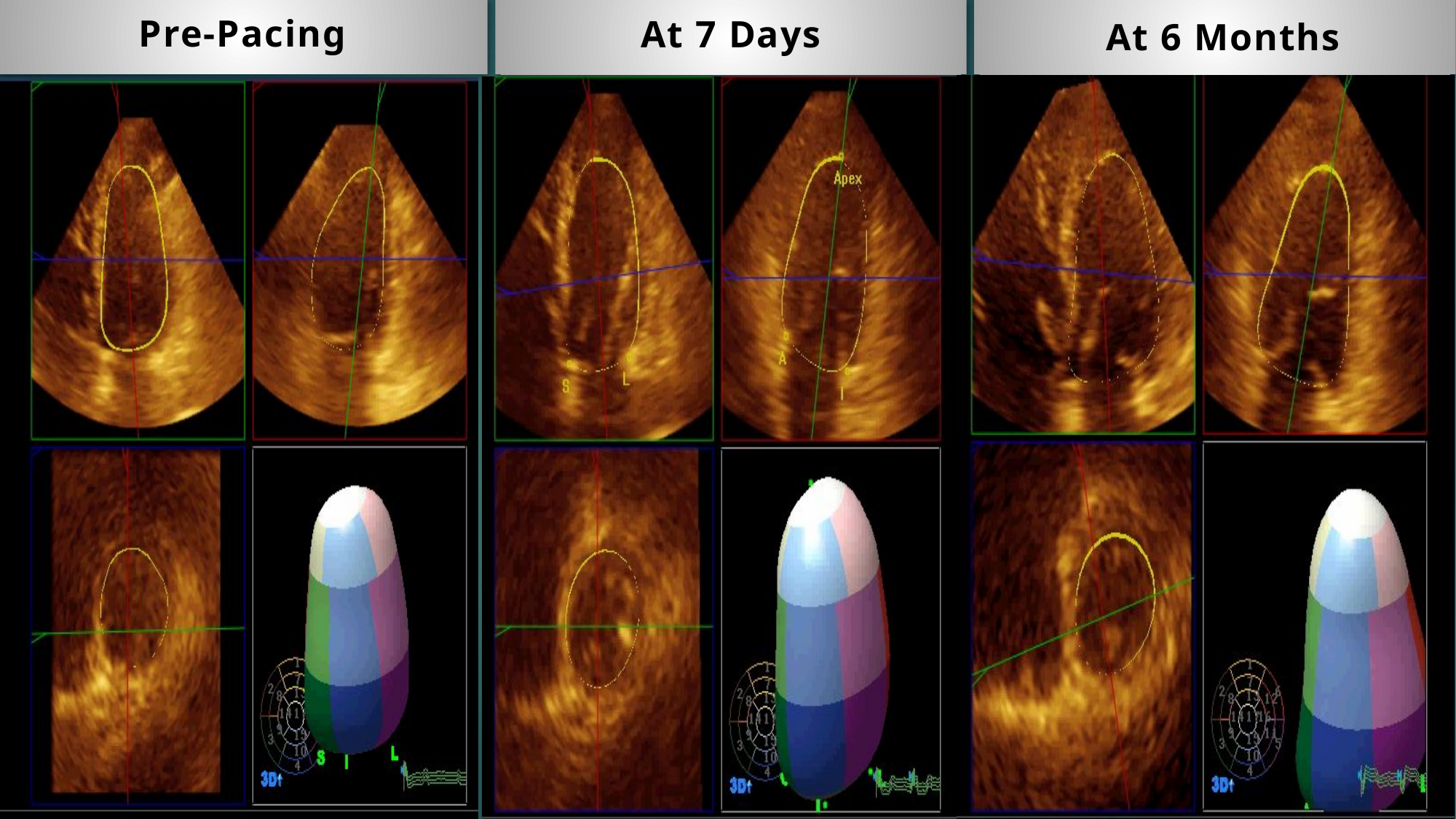

Pre-Pacing
At 7 Days
At 6 Months

## Slide 4
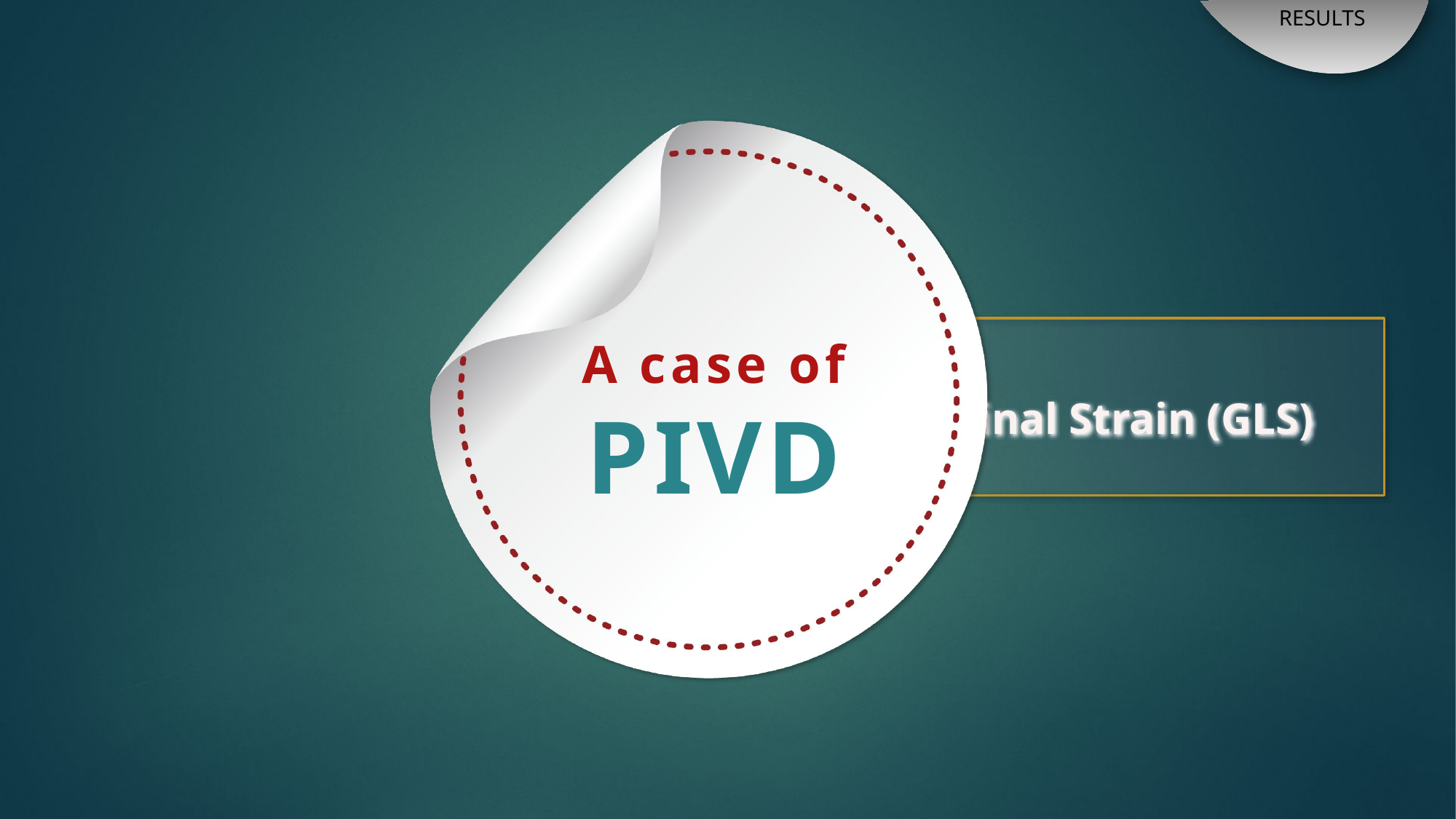

Results
A case of
PIVD
Global Longitudinal Strain (GLS)

## Slide 5
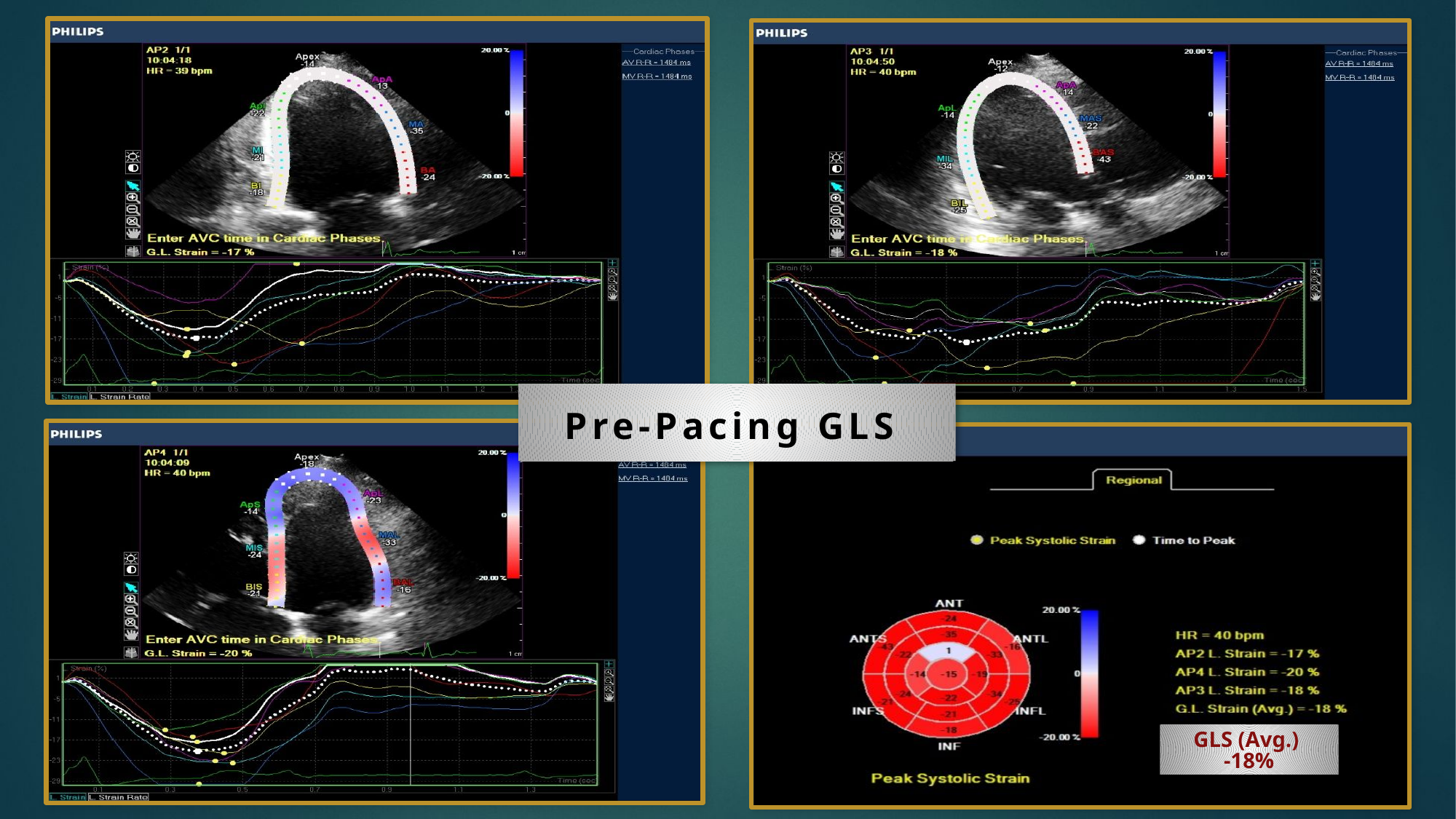

Pre-Pacing GLS
GLS (Avg.)
-18%

## Slide 6
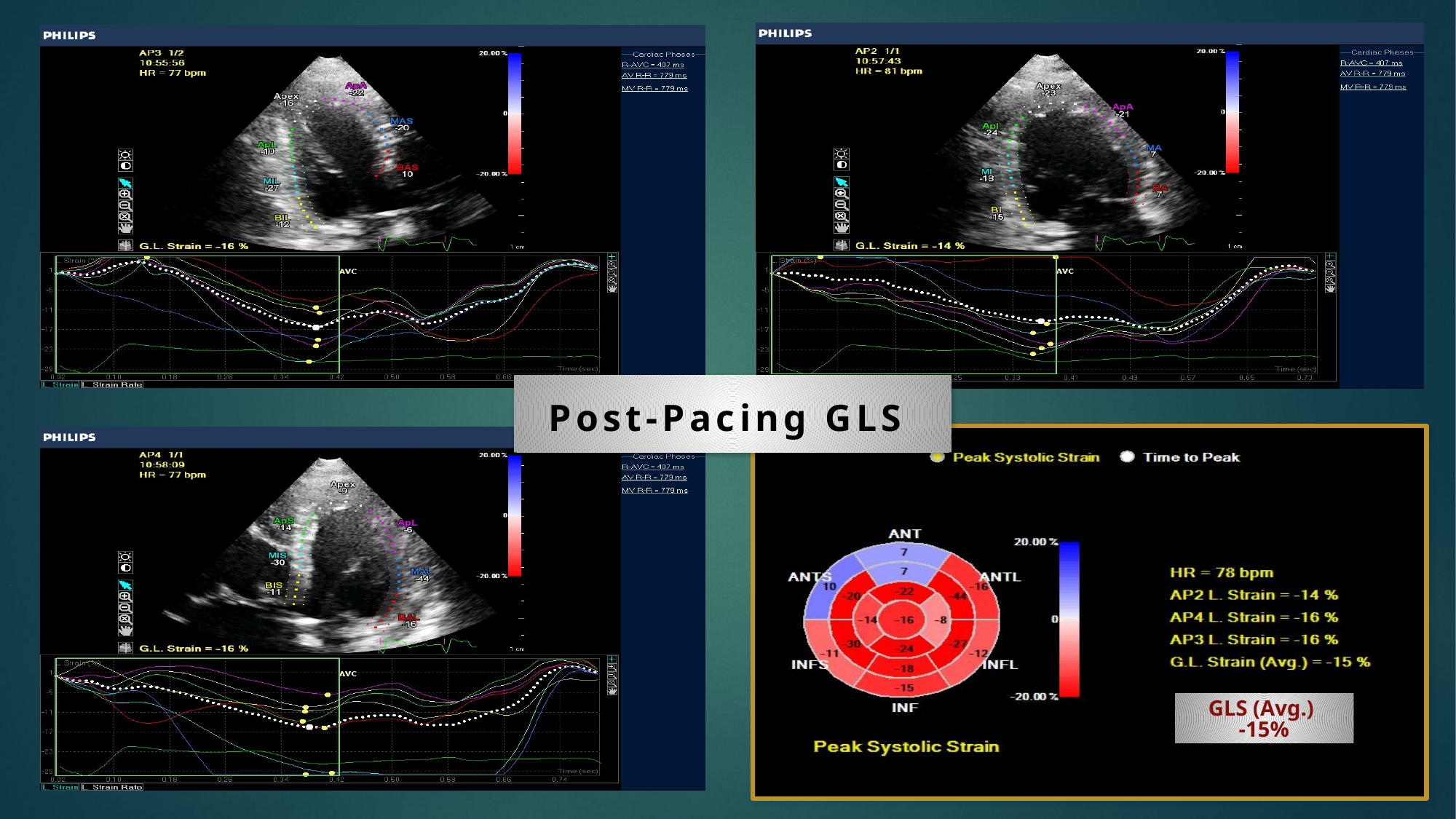

Post-Pacing GLS
GLS (Avg.)
-15%

## Slide 7
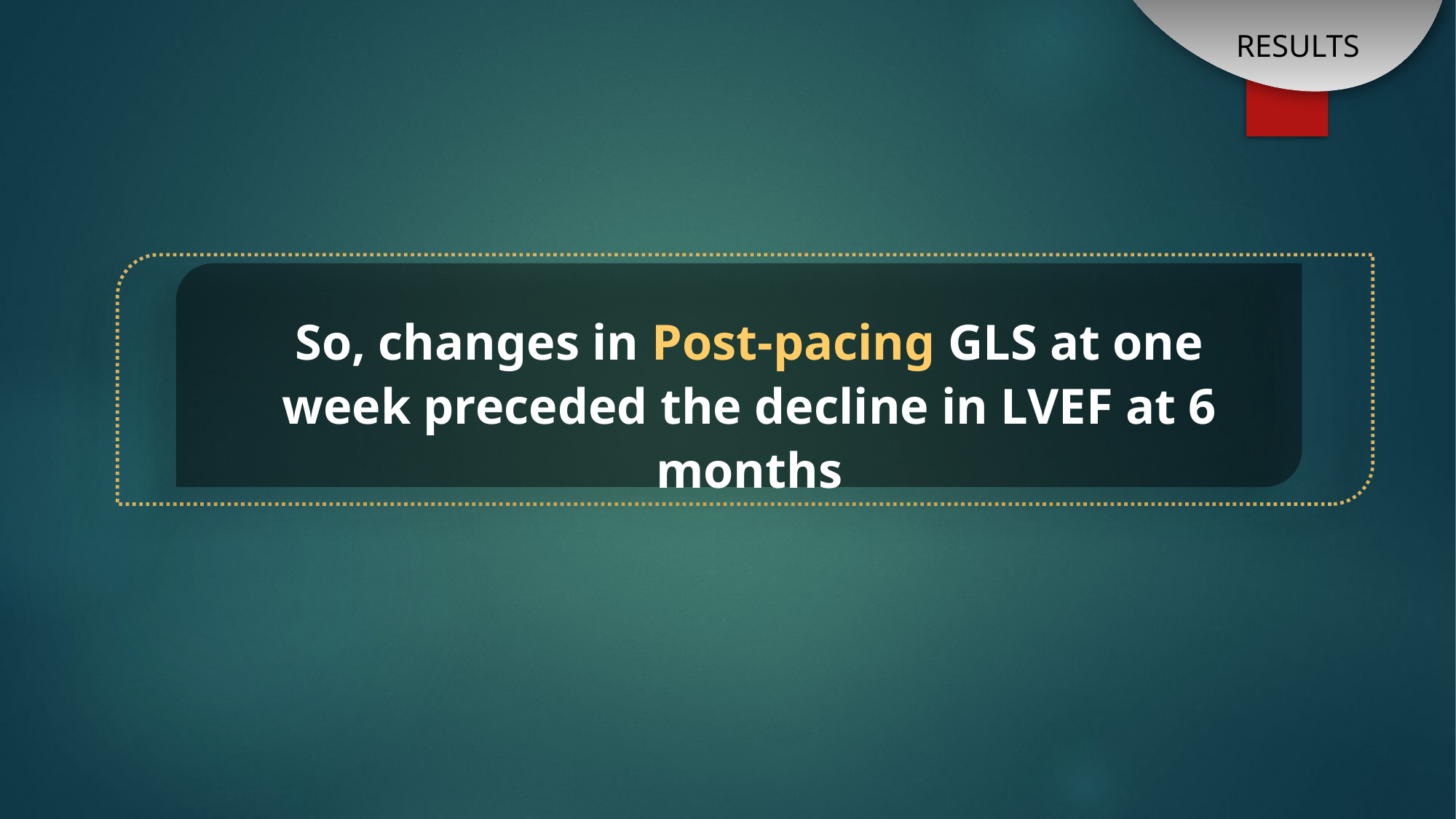

Results
So, changes in Post-pacing GLS at one week preceded the decline in LVEF at 6 months
