# Supplementary material for: Predictors of pacing induced left ventricular dysfunction and cardiomyopathy assessed by three-dimensional echocardiography and speckle tracking strain
Source: Egypt Heart J. 2021 Jan 26;73:10. doi: 10.1186/s43044-021-00136-x (PMC7838225; doi:10.1186/s43044-021-00136-x)
Supplement: Supplementary file 3 — Additional file 3. PIVD case 1 [file 43044_2021_136_MOESM3_ESM.pptx]

## Slide 1
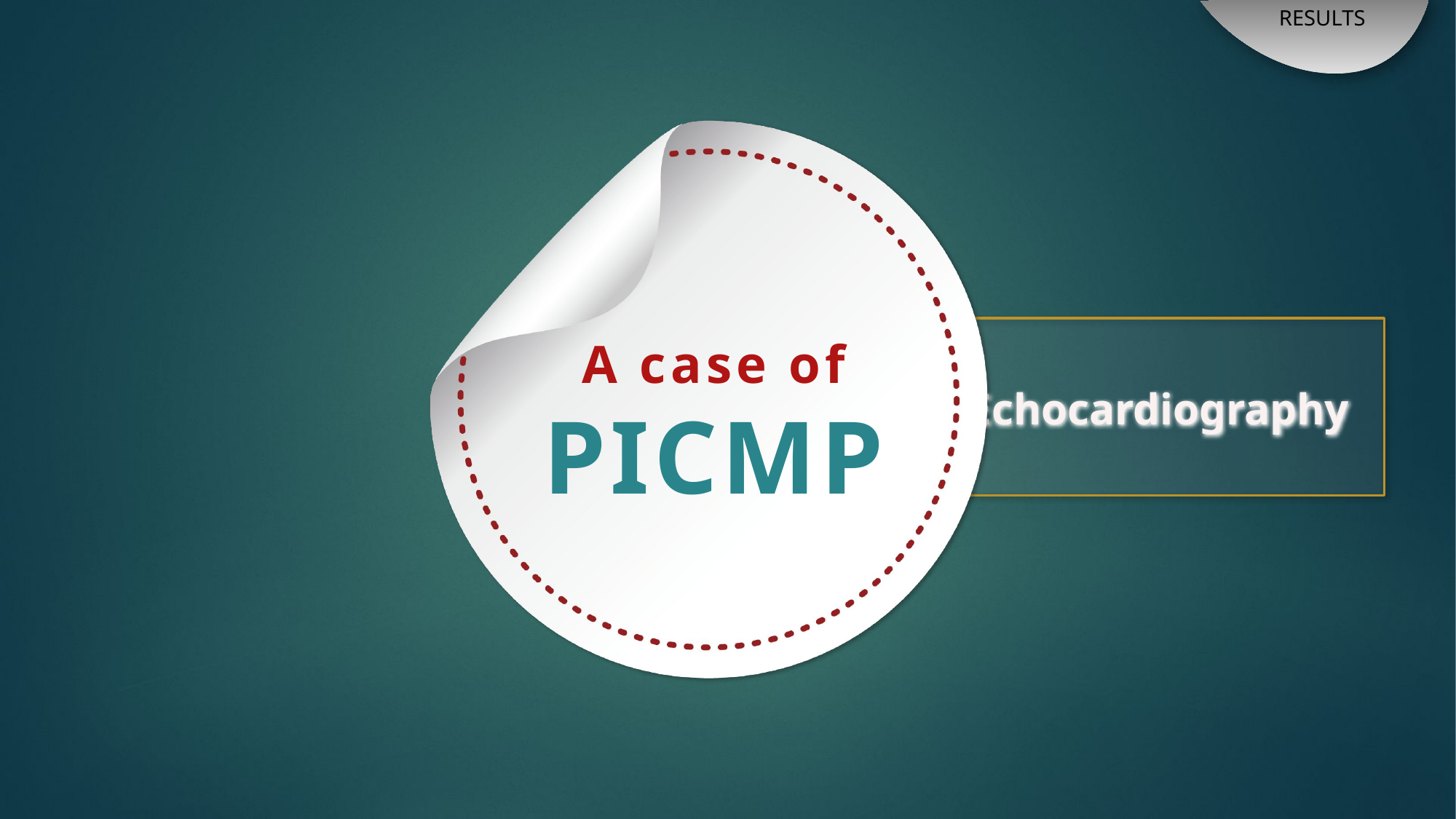

Results
A case of
PICMP
Full Volume 3D Echocardiography

## Slide 2
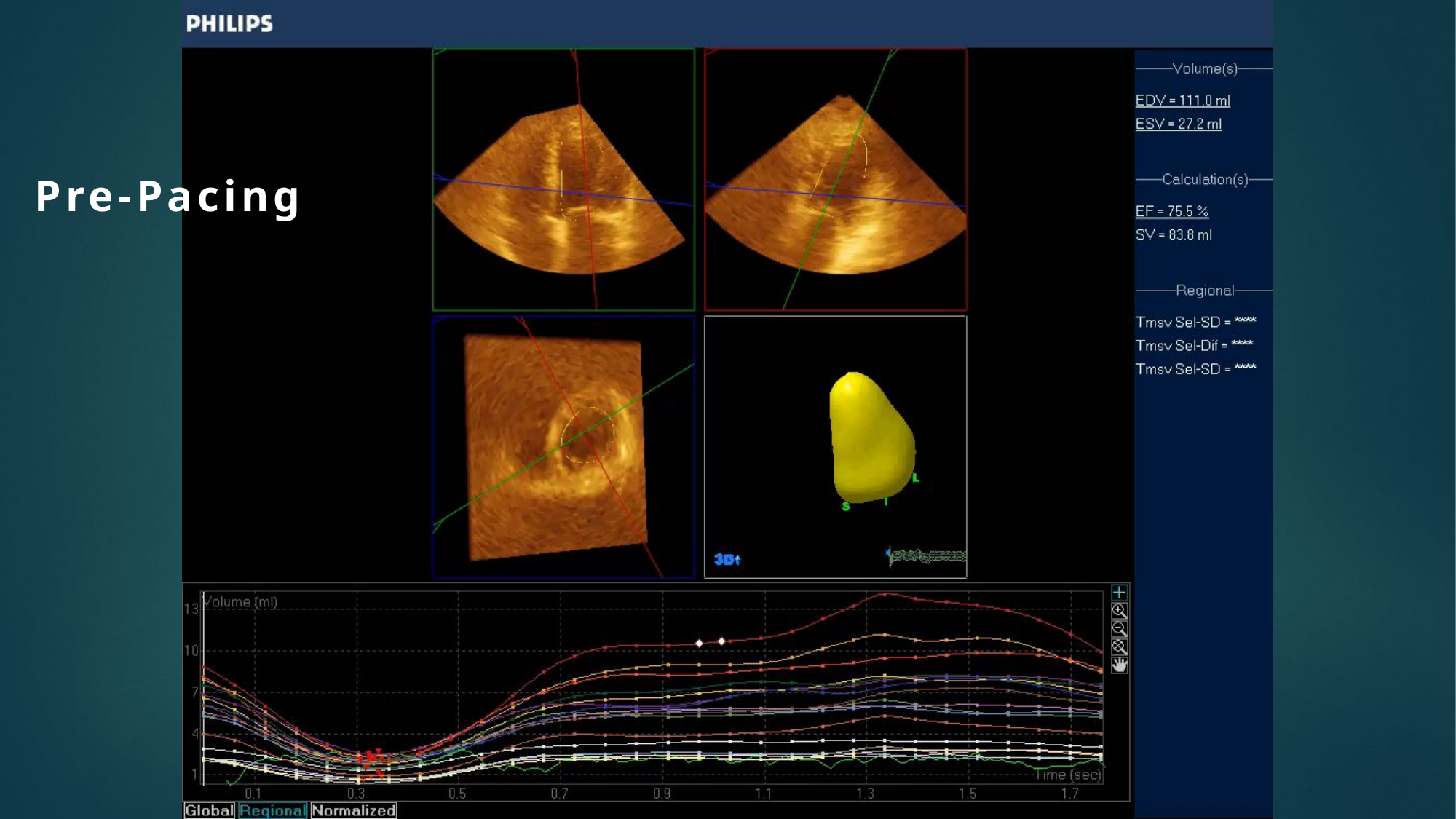

Pre-Pacing

## Slide 3
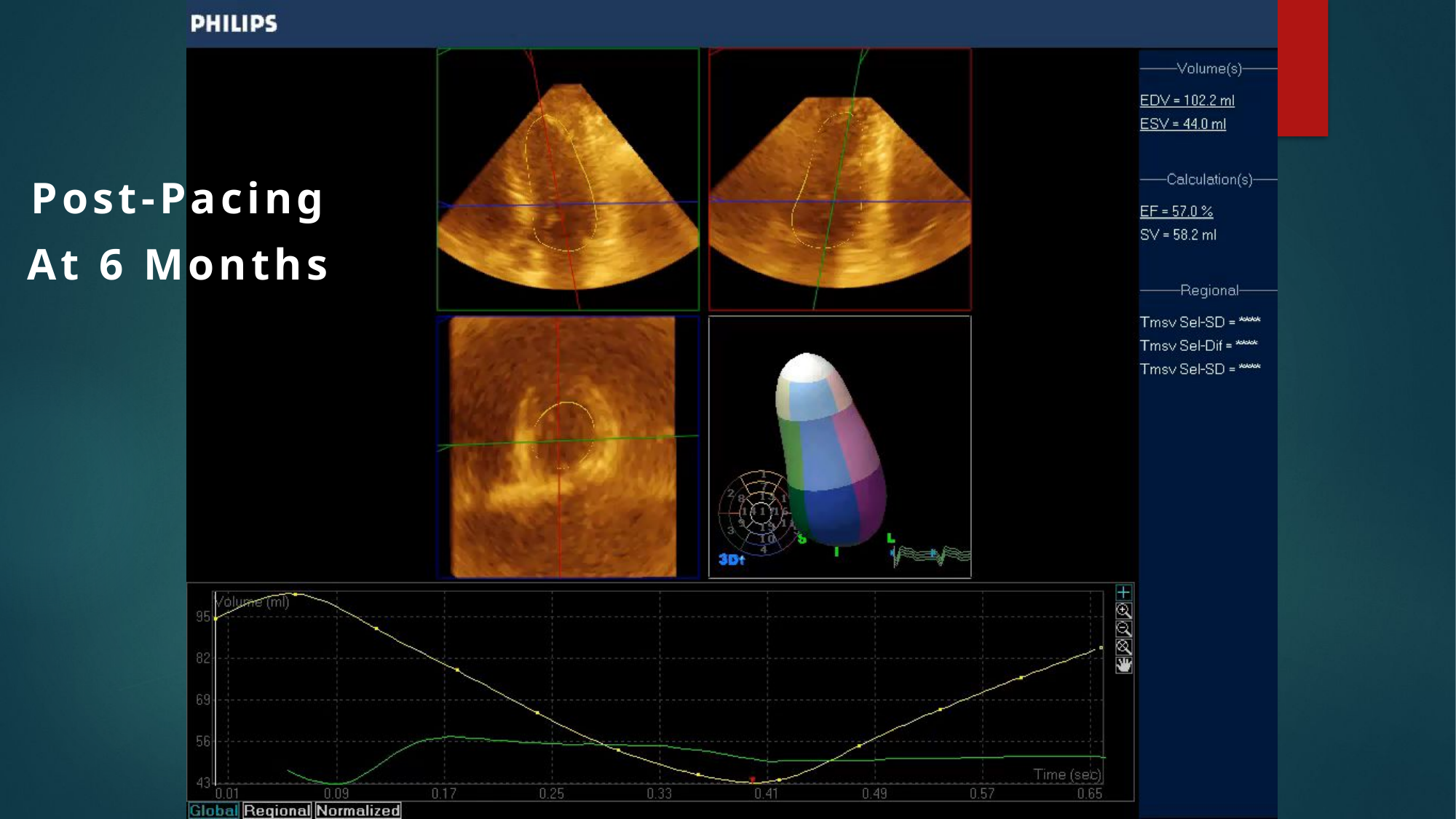

Post-Pacing
At 6 Months

## Slide 4
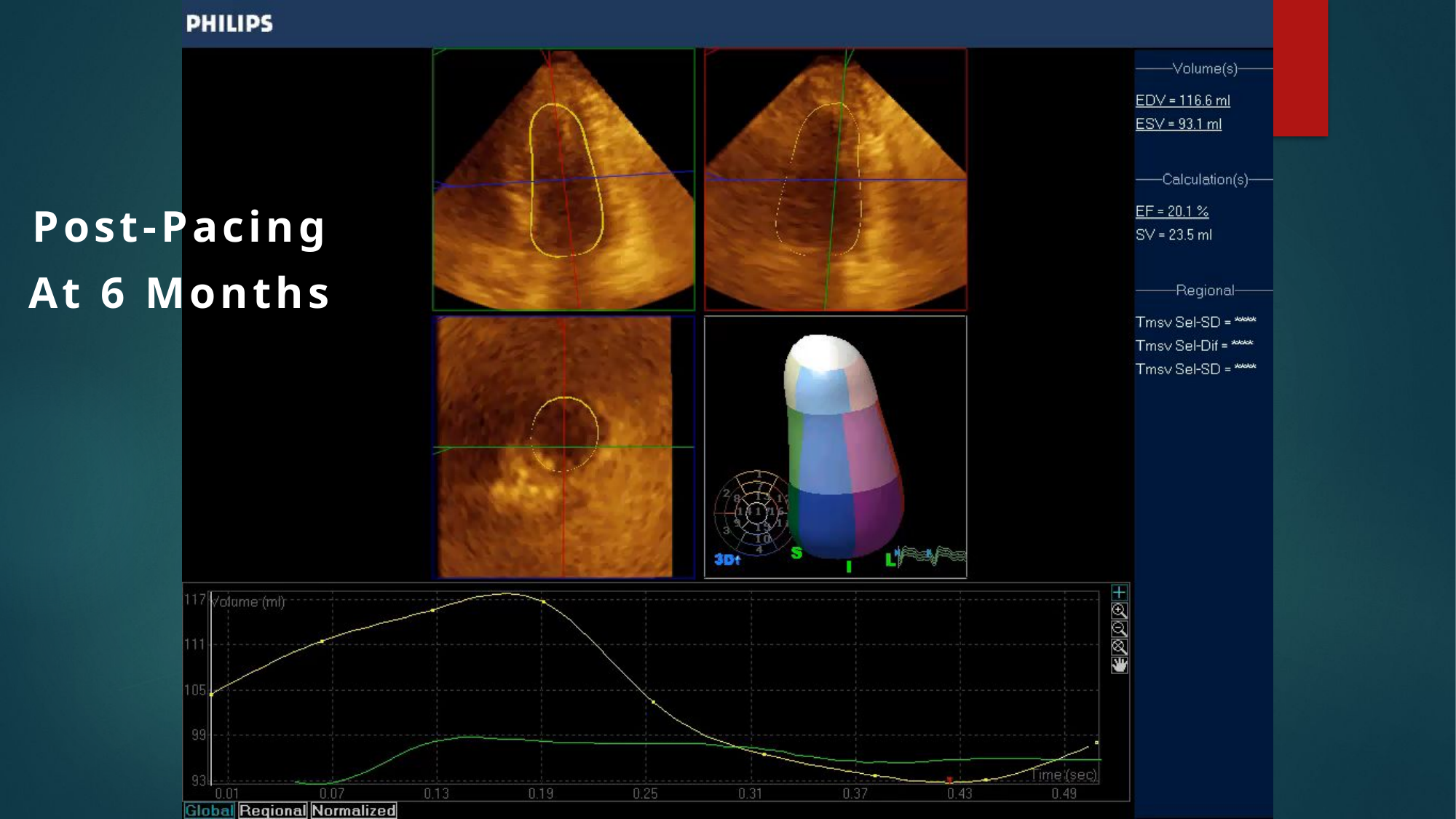

Post-Pacing
At 6 Months

## Slide 5
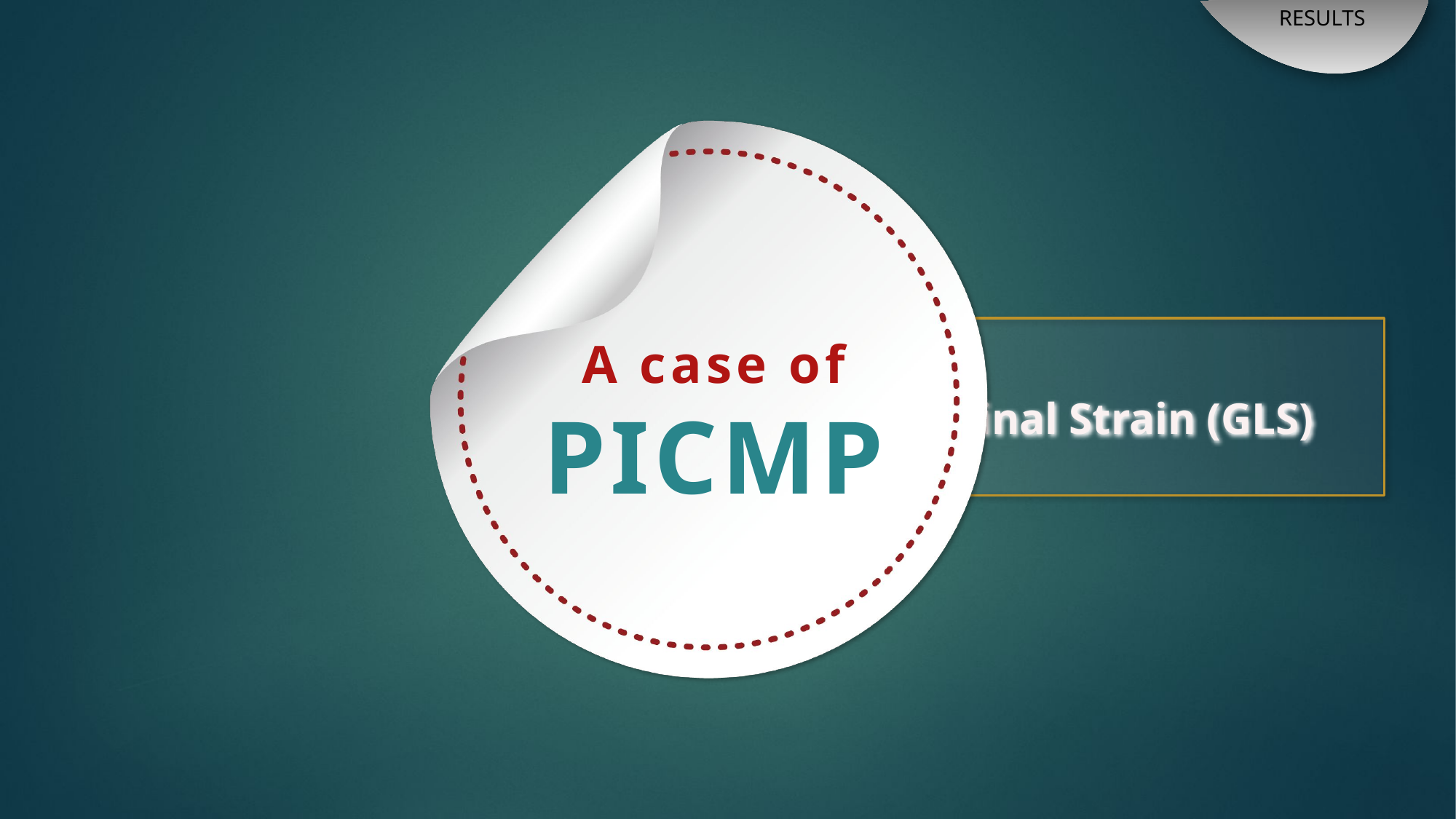

Results
A case of
PICMP
Global Longitudinal Strain (GLS)

## Slide 6
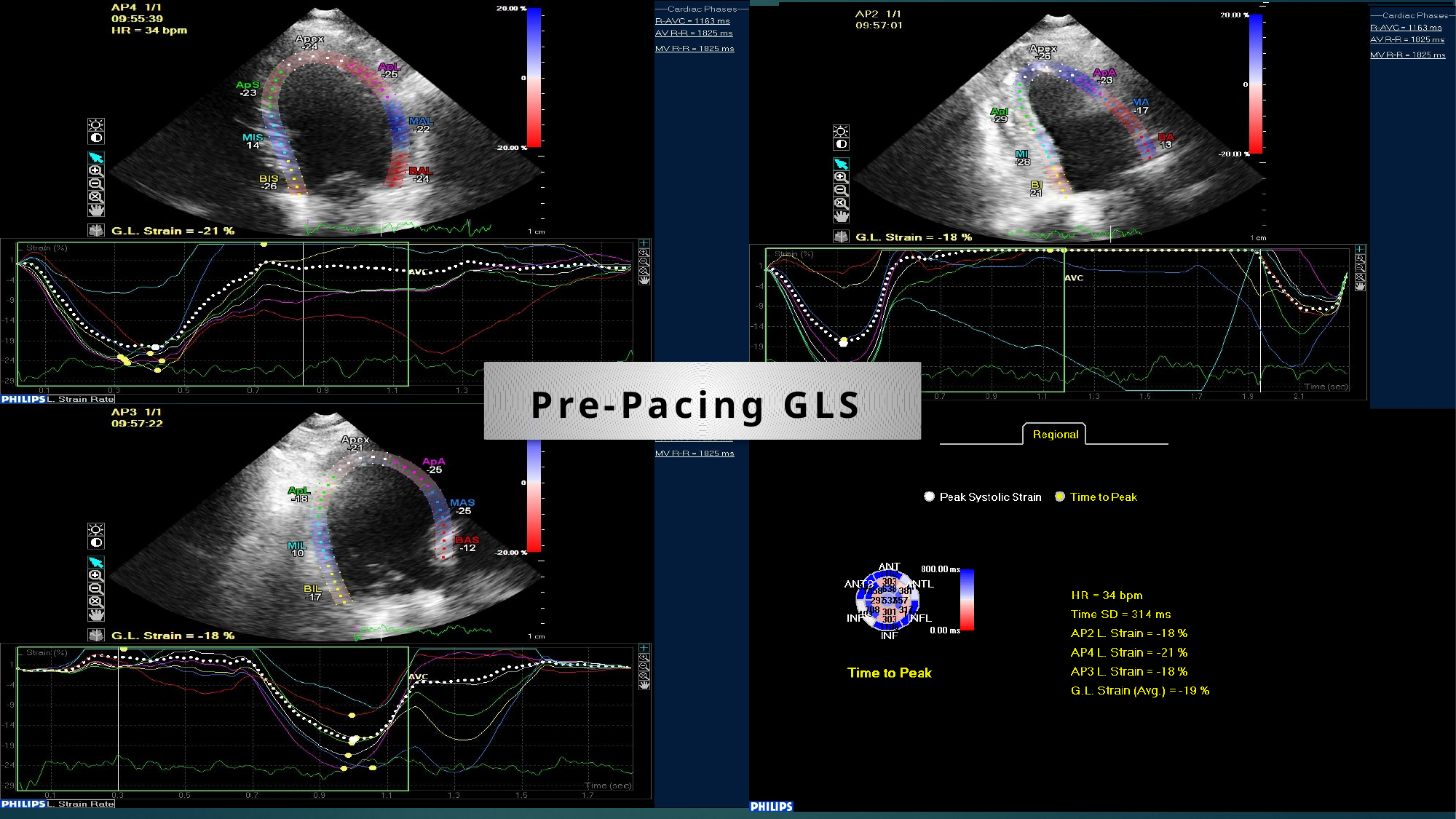

Pre-Pacing GLS

## Slide 7
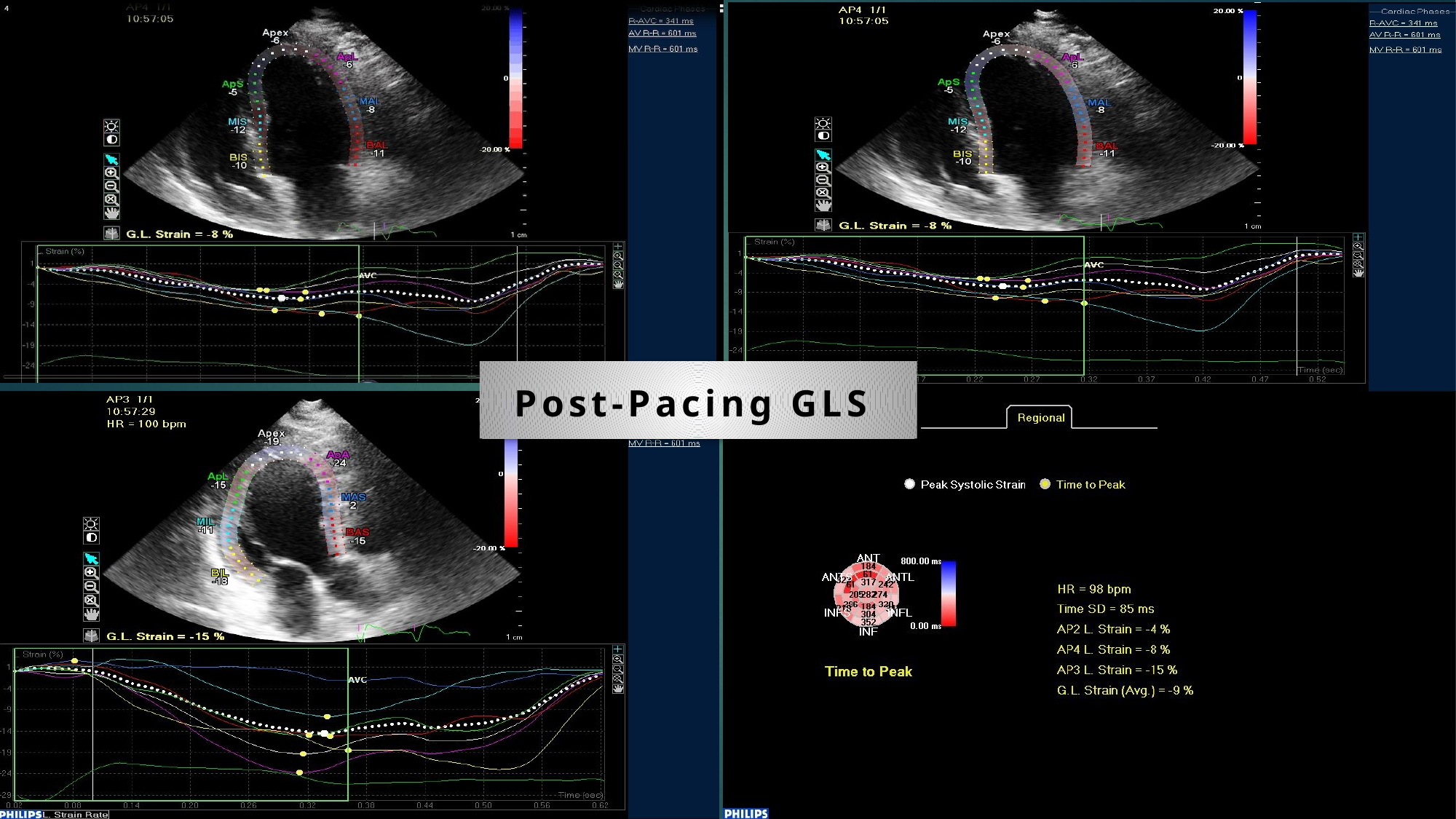

Post-Pacing GLS

## Slide 8
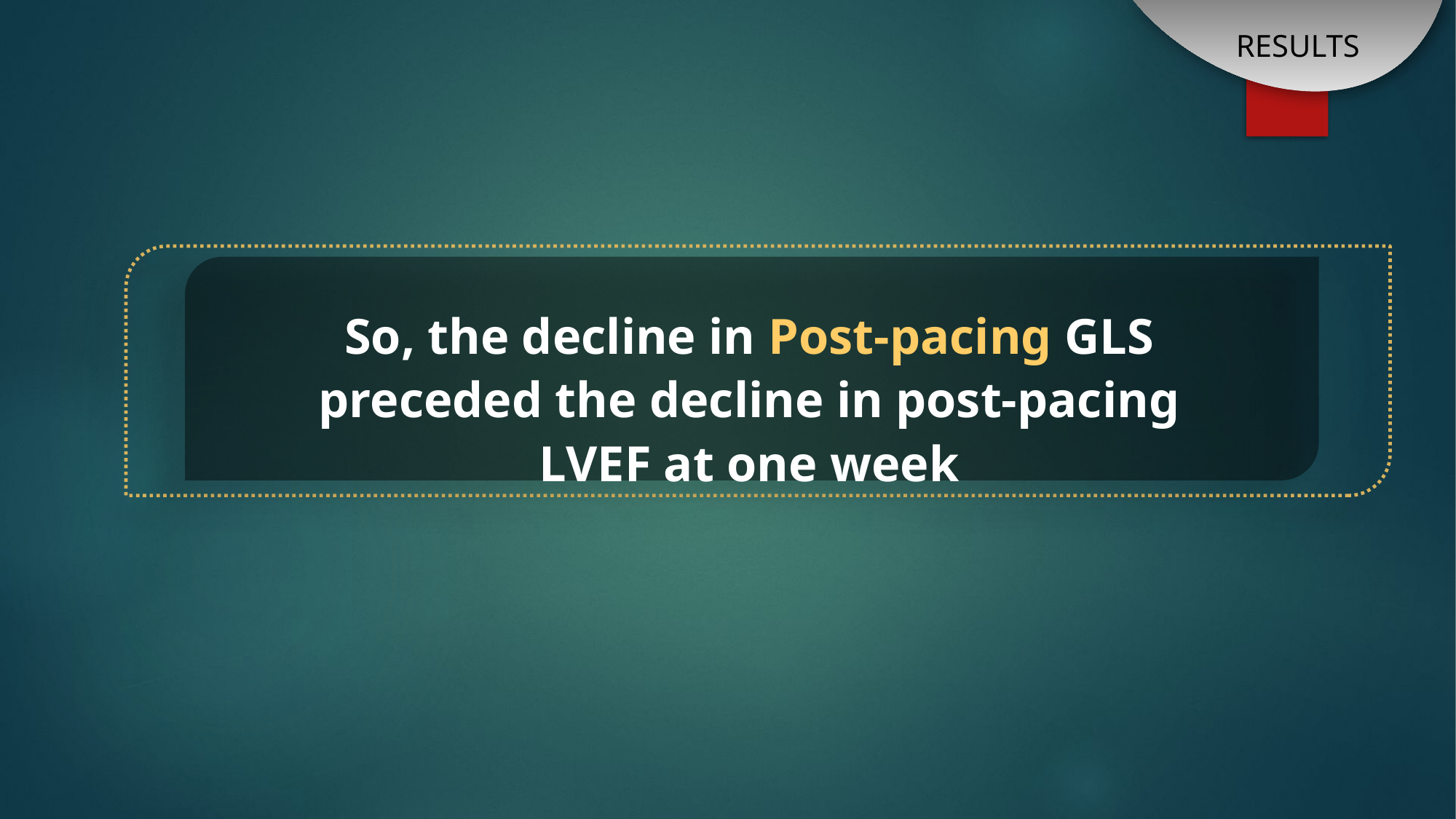

Results
So, the decline in Post-pacing GLS preceded the decline in post-pacing LVEF at one week
